# Predicting hand washing, mask wearing and social distancing behaviors among older adults during the covid-19 pandemic: an integrated social cognition model

**DOI:** 10.1186/s12877-022-02785-2

**Published:** 2022-02-02

**Authors:** Yanping Duan, Borui Shang, Wei Liang, Zhihua Lin, Chun Hu, Julien Steven Baker, Yanping Wang, Jiali He

**Affiliations:** 1 Department of Sport, Physical Education and Health, Faculty of Social Sciences, Hong Kong Baptist University, 12/F, Hong Kong Baptist University ShekMun Campus, 8 On Muk Street, ShekMun, Shatin, Hong Kong; 2 Centre for Health and Exercise Science Research, Hong Kong Baptist University, Hong Kong, China; 3 Department of Health Sciences, Wuhan Institute of Physical Education, Wuhan, China; 4 Department of Social Sciences, Hebei Sport University, Shijiazhuang, China; 5 Sport Section, Wuhan University, Wuhan, China; 6 Student Mental Health Education Center, Northwestern Polytechnical University, Xian, China

**Keywords:** COVID-19, Older adults, Hand washing, Mask wearing, Social distancing, Integrated Social Cognition Model

## Abstract

**Background:**

Older adults are at a higher risk from COVID-19. Individual preventive behaviors including frequent hand washing, mask wearing, and social distancing play important roles in reducing the transmission of COVID-19 in the community. This study aimed to identify the determinants of three preventive behaviors of older adults during the COVID-19 pandemic by using an Integrated Social Cognition Model.

**Methods:**

Using a prospective study design, 516 Chinese older adults from Hubei province of China (mean age = 67.55 years, SD = 6.60, 57.9% females) completed two online questionnaire surveys. The demographics, social cognition constructs (motivational self-efficacy, risk perception, attitude, subjective norm, health knowledge, intention, volitional self-efficacy, planning, action control) and three preventive behaviors were measured during the first-wave online survey from 18 May 2020 to 7 June 2020. One month later, three preventive behaviors were measured again during the second-wave online survey. Data were analyzed by structural equation modelling.

**Results:**

Models showed attitude, motivational self-efficacy and subjective norm were consistent predictors of intention, motivational self-efficacy was a consistent predictor of volitional self-efficacy, planning and volitional self-efficacy were consistent predictors of action control, and health knowledge was a consistent predictor of behaviors across all three preventive behaviors. In addition, mediating relationships were found in the model of hand washing behavior. In particular, planning (β = .109, *p* = .042) and action control (β = .056, *p* = .047) mediated between volitional self-efficacy and hand washing respectively. Action control also mediated between planning and hand washing (β = .087, *p* = .044). Moreover, the inclusion of past behaviors in three models attenuated most of the structural relations.

**Conclusions:**

The current study’s findings basically supported the Integrated Social Cognition Model and identified key modifiable determinants of preventive behaviors. Based on this model, future interventions aiming to promote COVID-19 preventive behaviors among older adults are warranted.

**Supplementary Information:**

The online version contains supplementary material available at 10.1186/s12877-022-02785-2.

## Background

The novel coronavirus disease 2019 (COVID-19) pandemic has become a truly global public health crisis since December 2019. As a vulnerable population group, older adults have suffered the most, accounting for nearly 75% of the COVID-19 relevant mortality globally [1, 2]. It has been advocated by many health authorities that the transmission of COVID-19 can be reduced by performing individual preventive behaviors including hand washing (HW), mask wearing (MW) and social distancing (SoD) [3, 4]. A recent policymaker’s guide also emphasized the effectiveness of compliance with the “3W’s” (Wash your hands, Wear a mask and Watch your distance/keep social distancing) during the COVID-19 prevention campaign [5]. In addition, public health organizations have been striving to develop behavioral interventions to promote these three preventive behaviors among the general population [6]. Given the above, identifying determinants of preventive behaviors that are potentially modifiable by interventions is an efficacious approach to promote the enactment of such behaviors.

Studies investigating preventive behaviors and their determinants in the general population have developed during the COVID-19 pandemic [7–9]. However, most of the studies targeted only one or two preventive behaviors and limited studies have addressed all three preventive behaviors simultaneously. This research area is even more limited among older adults [7–11]. To fill this evidence gap, the current study applied an integrated social cognition model to identify the determinants of HW, MW and SoD, and the processes involved, among Chinese older adults during the COVID-19 pandemic. The present study is expected to provide evidence of potentially modifiable variables for behavior change interventions aimed at promoting preventive behaviors among older adults. Such interventions may contribute to the alleviation of the infection rates in older adults during the COVID-19 pandemic, and further assist in preventing potential communicable disease pandemics in the future.

### Determinants of preventive behaviors: an integrated social cognition model

It has been a long tradition to apply social cognition approach to examine the determinants of health behaviors. As a classical social cognition theory, the TPB demonstrates that intention is the most proximal predictor of behavior [12]. Intention is a function of three constructs including attitude (positive or negative evaluations towards the consequences of performing the intended behavior), subjective norm (perceived expectations of significant others approving the intended behavior), and perceived behavioral control (PBC; beliefs in capability to perform the intended behavior) [12]. Intention is proposed to mediate the effects of attitude, subjective norm, and PBC on behavior. Previous research has found support for the TPB constructs in predicting the intentions and behaviors including hand hygiene [13, 14] and face mask wearing [15] across diverse populations.

Although the TPB constructs are parsimonious and appealing, there are still some limitations, such as inconsistent explanation, power of attitude, subjective norm, PBC on the intention [16], and the modest effect size of intention on behavior (implying the intention-behavior gap) [17]. Researchers have therefore suggested introducing additional factors from other theories to the TPB to predict intention and behavior more effectively focusing on the intention behavior gap [18]. For example, health knowledge (the knowledge about a disease’s causes and consequences and its prevention) had been added to the prediction model regarding intention of oral health behavior [19]. Moreover, health knowledge can directly predict hand washing behaviors among diverse populations [20, 21] as well as mask wearing and social distancing among older adults during the COVID-19 pandemic [22–24]. Additionally, recent studies applying the Health Action Process Approach (HAPA) [25] to the preventive behaviors suggested that motivational self-efficacy (Motivational SE; the beliefs about the ability to start the behavior) can significantly predict the intention of mask wearing and the intention of hand washing [26]. Risk perception (e.g., perceived susceptibility and severity of certain health threat), as one social cognitive factor of HAPA, was an important predictor of the intention toward social distancing [27] and the intention toward all preventive behaviors [28]. To optimize the explanation power of TPB constructs on the intention and behaviors, we therefore included health knowledge, Motivational SE and risk perception as additional predictors of intention. Meanwhile, health knowledge was added to the TPB as a predictor of behaviors.

To resolve the limitation of the intention-behavior gap existing in the TPB and the extended TPB, the HAPA proposes that individuals need to augment their intentions with a range of self-regulatory strategies in order to enact behavior. For example, the self-regulatory strategy of planning is part of the process of intention enactment. Intentions are more likely to be translated into action when people plan when, where, and how they will perform the intended behavior (action planning) and how to overcome barriers to its achievement (coping planning) [25]. Planning mediates the relationship between intention and behavior, as shown in previous studies [29, 30]. While planning is a prospective strategy, suggesting behavioral plans are made before the situation is encountered, action control is a concurrent self-regulatory strategy, where the ongoing behavior is constantly evaluated. Action control is a three-facet construct comprising: self-monitoring, awareness of standards and self-regulatory effort [31]. Previous evidence has shown that action control can mediate between intention and facemask wearing [26].

HAPA also proposes self-efficacy beliefs as important self-regulatory strategies and are phase-specific, thus several types of self-efficacy can be distinguished [25]. Motivational SE is contributory to intention formation during the pre-intentional phase. Volitional self-efficacy (Volitional SE), on the other hand, is a group of optimistic beliefs during the post-intentional phase, including maintenance self-efficacy (beliefs about the ability to persevere the behavior in the face of obstacles) and recovery self-efficacy (beliefs about the ability to re-start the new behavior after the disengagement) [25]. Volitional SE can mediate the effect of Motivational SE on hand washing behavior, as shown in many studies [32, 33]. In addition, according to the HAPA, there are certain mediation relationships among Volitional SE, planning and action control when they predict behaviors [25]. For example, planning and action control can mediate the effects of Volitional SE on health behaviors respectively [34, 35], and action control can mediate the effects of planning on health behavior [36].

Although the TPB and the HAPA emphasize the importance of social cognition process of health behaviors, research applying those theories has shown that past behavior remains a pervasive determinant of behavior alongside the theory constructs [10, 14]. The inclusion of past behavior as an independent behavioral determinant in a social cognition theory is significant as it provides a test of its sufficiency in explaining unique variance in behavior.

### The present study and hypotheses

The present study aimed to identify the determinants of participation in three preventive behaviors (HW, MW and SoD) among Chinese older adults during the COVID-19 pandemic using an Integrated Social Cognition Model. This model incorporated the TPB constructs (attitude, subjective norm, PBC and intention), health knowledge and the HAPA constructs (risk perception, Motivational SE, intention, Volitional SE, planning and action control). In some research, Motivational SE in the HAPA model was regarded as a synonymous construct with PBC in TPB theory [37]. Therefore, to keep the parsimony of the integrated model, we merged the PBC in the TPB to the Motivational SE in the HAPA. As a result, the integrated model consisted of 9 social cognitive constructs of behavior (attitude, subjective norm, motivational SE, risk perception, health knowledge, intention, volitional SE, planning and action control). The above approach rides on the cusp of recent research applying integrated theoretical models [10]. We examined predictions of the integrated model in a two-wave prospective survey.

**Fig. 1:**
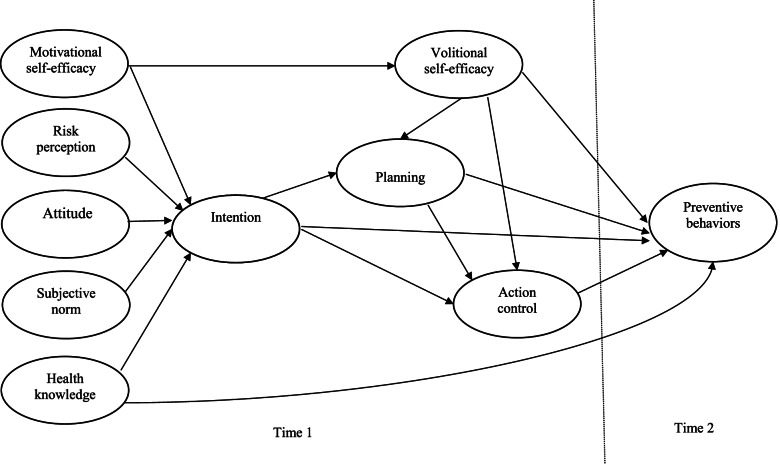
The hypothesized Integrated Social Cognition Model based on knowledge, Theory of Planned Behavior (TPB) and Health Action Process Approach (HAPA) in predicting COVID-19 preventive behaviors among older adults. Note. All hypothesized relationships are positive in direction

In total, 16 direct paths were hypothesized in the Integrated Social Cognition Model for each preventive behavior (see Fig. 1). During the pre-intentional phase, it was expected that risk perception, attitude, subjective norm, Motivational SE, and health knowledge (Time 1) would predict intention (Time 1); intentions and health knowledge (Time 1) would predict behavior (Time 2); and Motivational SE (Time 1) would predict Volitional SE (Time 1). After the intention had been formed, it was expected that intention (Time 1) would predict planning and action control (Time 1); Volitional SE (Time 1) would predict planning, action control (Time 1) and behavior (Time 2); planning would predict action control (Time 1); and planning and action control (T1) would predict behavior (Time 2) (Hypothesis 1).

In total 11 indirect mediating relationships in the Integrated Social Cognition Model for each preventive behavior were hypothesized. It was expected that intention would mediate effects of Motivational SE, risk perception, attitude, subjective norm and health knowledge (Time 1) on behavior (Time 2) respectively. Volitional SE (Time 1) would mediate effects of Motivational SE (Time 1) on behavior (Time 2), planning and action control (Time 1) would mediate effects of intention (Time 1) on behavior (Time 2) respectively. It was also expected that planning and action control (Time 1) would mediate effects of Volitional SE on behavior (Time 2) respectively, and action control (Time 1) would mediate effects of planning (Time 1) on behavior (Time 2) (Hypothesis 2).

We further examined the model including past behavior (Time 1) as a direct predictor of all social cognition constructs and behavior. It was expected that significant effects of past behavior on all constructs in the model would occur, which attenuates the strength of relationships between social cognition constructs and behaviors (Hypothesis 3).

## Methods

### Study design and participants

This study adopted a prospective design comprising a two-wave online survey. With a snowball sampling strategy, the first-wave survey was conducted from 18 May 2020 to 7 June 2020, which is around one to two months after the lockdown was withdrawn in the Hubei province of China. The second-wave survey was launched at one-month follow-up. We recruited 667 Chinese older adults (56.7% female) from five cities in Hubei, China, including Wuhan, Xiaogan, Jingzhou, Shiyan, and Xiangyang to complete the first-wave survey. All recruited participants were community-dwelling older adults (≥ 60 years) and met the eligibility criteria: 1) had not been infected with COVID-19; 2) do not have any cognitive disorders; 3) have access to a mobile phone or laptop; and 4) are proficient in reading or listening in Chinese. For those participants who had difficulty in using a mobile phone, their family members or friends were invited to assist them to complete the survey. Baseline sample characteristics were presented in the baseline section of Table 1.

**Table 1:** Sample characteristics and descriptive statistics for study variables at baseline and 1-month follow-up

Variable	Baseline	Follow-up
**Number**	667	516
**Age**		
Range (years old) Mean (SD)	60-89 67.43 (6.52)	60-89 67.55 (6.60)
**Gender**, n (%)		
Male Female	289 (43.3) 378 (56.7)	217 (42.1) 299 (57.9)
**Marital status**, n (%)		
Single Married Divorced or widowed	11 (1.6) 571 (85.7) 85 (12.7)	14 (2.7) 432 (83.7) 70 (13.6)
**Living situation**, n (%)		
Alone With spouse/partners/ Children	58 (8.7) 609 (91.3)	48 (9.3) 468 (90.7)
**Education level**, n (%)		
Primary school or below Middle or high school College or above	68 (10.2) 301 (45.1) 298 (44.6)	45 (8.7) 231 (44.8) 240 (46.5)
**Professional status**, n (%)		
Unemployed without pension Pensioner or retired Part-time or full-time employment	44 (6.6) 596 (89.4) 27 (4.0)	22 (4.3) 478 (92.6) 16 (3.1)
**Economic situation**, n (%)		
Below average Average Above average	153 (22.9) 380 (57.0) 134 (20.1)	113 (21.9) 299 (57.9) 104 (20.2)
**Chronic disease situation**, n (%)		
Yes No	330 (49.5) 337 (50.5)	262 (50.8) 254 (49.2)
**Subjective health status**, n (%)		
Bad Satisfactory Excellent	59 (8.8) 263 (39.4) 345 (51.7)	48 (9.3) 196 (38.0) 272 (52.7)
**Infected cases of acquaintances**, n (%)		
Yes No	59 (8.8) 608 (91.2)	50 (9.7) 466 (90.3)
**BMI (kg/m**^**2**^**)**, mean (SD)	23.10 (2.66)	23.06 (2.67)
< 18.5, n (%) 18.5 ≤ BMI < 23, n (%) 23 ≤ BMI < 26, n (%) ≥ 26, n (%)	23 (3.4) 292 (43.8) 271 (40.6) 81 (12.1)	19 (3.7) 228 (44.2) 206 (39.9) 63 (12.2)

Note: *SD* Standard Deviation

### Procedures

The survey was constructed and administered using an online survey platform in China, namely SOJUMP (Changsha Ranxing Information Technology Co., Ltd., China). All recruitment posters and the hyperlink for the survey were disseminated via a mobile Short Message Service (SMS) and popular social media platforms in China (e.g., WeChat, Weibo, and QQ). Three approaches were used for recruiting participants: 1) Relying on the researchers’ personal social networks in five cities of Hubei province, the eligible family members, friends and relatives of the researchers were invited. The participants then encouraged their friends to attend the survey; 2) Researchers contacted the directors of community neighborhood committees in Wuhan and Xiaogan, respectively and sought their collaboration and support. Upon receiving the agreement of directors, researchers were permitted to enter into their community neighborhood WeChat groups to recruit eligible participants; 3) Researchers contacted officials who were in charge of the retirement in two universities in Wuhan of Hubei Province. With the support of officials, a recruitment poster and survey hyperlink were delivered to their internal WeChat group, especially for retirement colleagues.

To boost the engagement of participation, each participant who completed two-wave surveys was offered 50 RMB incentive by electronic transfer via WeChat or Alipay or by prepaid telephone recharge. All participants were asked to sign an informed consent form on the first page of the survey platform before completing the questionnaires. Ethical approval for the study was obtained from the Research Ethics Committee of Hong Kong Baptist University (REC/19-20/0490).

### Measurement

The demographic characteristics included age, gender, marital status, living situation, education level, professional status, economic situation, chronic disease situation. Participants were also invited to report their self-evaluated health status, infected cases of acquaintances, body height (cm) and body weight (kg).

Behavioral variables (hand washing, mask wearing, and social distancing) [38] and social-cognitive variables including risk perception [39], health knowledge [40], attitude [41], subjective norm [15], intention [42, 43], motivational self-efficacy [42, 43], volitional self-efficacy [42, 43], planning [42, 43], and action control [14] were measured by questionnaires. The details of the measurement tools are outlined in Table 2.

**Table 2:** Overview of the measurement tools regarding behavioral and social cognitive variables

Name of Variable	Origin	Detailed Information
*Behavioral Variables*		
Hand Washing	Liang et al., 2021 [38]	2 items; 4-point Likert scale from (1) never to (4) always; “During the previous week, how frequently did you wash your hands with soap and water or alcohol-based hand rub (for at least 20 seconds, all surfaces of the hands) … ”, followed by two kinds of situations, i.e., “in the daily life situations (e.g., before preparing food; before eating; after defecation)” or “in disease-related situations (e.g., after blowing nose, coughing, or sneezing; before and after caring for the sick)”
Mask Wearing	Liang et al., 2021 [38]	2 items; 4-point Likert scale from (1) never to (4) always; “During the previous week, I have usually worn a face mask properly … ” followed by two different situations relevant to older adults, i.e., “when visiting public places”, and “caring for a person with suspected COVID-19 infection”
Social Distancing	Liang et al., 2021 [38]	2 items; 4-point Likert scale from (1) never to (4) always; “a) stayed out of crowded places or mass gatherings when going outside of my home, and b) kept space (at least 1 meter) between myself and other people who were coughing or sneezing.”
*Social Cognitive Variables*		
Risk Perception	Duan et al., 2017 [39]	2 items; VAS ranging from 0 = lowest to 100 = highest; “How likely do you believe it is for you to become infected with COVID-19 if you do not wash hands frequently/wear face mask /keep a secure social distancing?”, and “Compared to an average person of your age and gender, what is your risk of COVID-19 infection from lack of frequent hand washing/face mask wearing/social distancing?”
Health Knowledge	Li & Liu, 2020 [40]	(At the beginning, clear instructions of the WHO recommendations for each of the three preventive behaviors were provided, e.g. “According to the WHO recommendations, the proper mask use consists of the following aspects, namely, when and how to wear face masks: 1) if you are taking care of a person with suspected 2019-nCoV infection; 2) if you are coughing or sneezing … ”.) 1 item; 4-point Likert scale from (1) do not know to (4) know all; “Have you known how and in what situations to wash hands/ wear face mask/ keep a secure social distancing in accordance with the WHO recommendations?”
Attitude	Rosen et al., 2009 [41]	4 items; VAS ranging from 0 = lowest to 100 = highest; a common stem on three preventive behaviors “For me to wash hands frequently/wear face mask/keep a secure social distancing during the outbreak of COVID-19 would be … ” followed by 4 bipolar sliders: harmful-beneficial, troubling-reassuring, unpleasant-pleasant, and optional-necessary.
Subjective Norm	Chung et al., 2018	2 items; VAS ranging from 0 = lowest to 100 = highest; “Most people who are important to me (e.g., my family members, friends, doctors) think that I should wash hands frequently/wear face mask/keep a secure social distancing during the outbreak of COVID-19”, and “Most people who are important to me wash hand frequently/wear face mask/keep a secure social distancing during the outbreak of COVID-19”
Intention	Duan et al., 2018; Liang et al., 2019	2 items; VAS ranging from 0 = lowest to 100 = highest; Sample items were “Today and in the near future, I intend to properly wash my hands in daily life situations/wear a face mask when I visit public places/stay out of crowded places or mass gatherings.
Motivational Self-efficacy	Duan et al., 2018; Liang et al., 2019 [39, 38]	2 items; VAS ranging from 0 = lowest to 100 = highest; “I feel certain that I can begin to wash my hands/wear my mask/keep social distance frequently even if it is time-consuming”, “I feel certain that I can begin to wash my hands/wear my mask/keep social distance even if it may cause inconvenience to my life”
Volitional Self-efficacy	Duan et al., 2018; Liang et al., 2019 [39, 38]	2 items; VAS ranging from 0 = lowest to 100 = highest; “I feel certain that I can maintain washing my hands frequently/wearing face mask/keeping a secure social distancing even if it takes much time for that to be part of my daily routine”. “I feel certain that I can restart to wash my hands frequently/wear face mask/keep a secure social distancing even if I forgot to do it a few times”
Planning	Duan et al., 2018; Liang et al., 2019 [39, 38]	2 items; VAS ranging from 0 = lowest to 100 = highest; “I have already made a concrete action plan regarding when, where and how to wash hand /wear face mask/keep social distancing” “I have made a coping plan to maintain frequent hand washing/mask wearing/social distancing if I am confronted with some barriers”
Action Control	Zhang et al., 2020 [14]	3 items; VAS ranging from 0 = lowest to 100 = highest; “I consistently monitor how and in what situations I wash my hands/wear my mask/keep social distance”, “I continuously make sure that I wash my hands/wear my mask/keep social distance properly”, and “I really try hard to wash my hands/wear my mask/keep social distance in necessary situations”

The demographic information and social cognition constructs of three preventive behaviors were measured at Time 1. The three preventive behaviors were measured at both Time 1 and Time 2. Each participant took 20-30 minutes to complete the first-wave online survey and 5-10 minutes to complete the second-wave online survey.

### Statistical analysis

Data was analyzed using SPSS 25.0. Missing data were imputed using EM algorithm multiple imputation analysis [44]. Kurtosis and skewness tests were used for data normality checks. Variables were considered non-normally distributed if they exceeded a kurtosis value larger than 7 or smaller than -7 and/or a skew value larger than 2 or smaller than -2 [45]. Descriptive statistics (frequency, percentage) of demographic variables were demonstrated. Independent sample T-tests were used to examine differences in demographics, social cognition constructs, and preventive behaviors between completers with two-wave data and drop-out sample with first wave data. Means, SD and internal consistency reliability (Cronbach’s α) of social cognition constructs as well as correlations among social cognition constructs and behavioral variables in the path analyses were examined using the Pearson test (two-tail, .05 as significance level).

Structural equation modelling (SEM) was used to test hypothesized integrated model for three preventive behaviors respectively. Fit indices, such as chi-square (χ^2^), the Comparative Fit Index (CFI), the Tucker-Lewis Index (TLI), the Standardized Root Mean Square Residual (SRMR) index, and the Root Mean Square Error of Approximation (RMSEA) index, were used to determine the hypothesized model’s fit. A non-significant χ ^2^ value (p > .05) is indicative of a path model that fits the data well [46]. Values of CFI and TLI range from 0 to 1, with higher values indicating better model fit (≥.90 indicated acceptable model fit, ≥.95 indicated good model fit). Smaller values of SRMR and RMSEA indicate more desirable models, with .08 or less indicating acceptable model fit and .05 or less suggesting good model fit. For all direct and indirect paths in the SEM, standardized coefficients (β) with 95% confidence intervals (CI) were calculated using maximum likelihood estimation and all significance levels were set as .05. In this study, we did not adjust for alpha-error inflation due to two considerations: 1) the current study was theory-driven rather than purely data-driven. It was suggested that multiplicity and Type-I error rate inflation are not a concern if only specific, prior hypotheses (i.e., these hypotheses are precise predictions of results and are based on well-developed theories) were examined [47]; 2) using adjusted approaches (e.g., Bonferroni) to control for the alpha-error inflation would magnify the Type-II error and decrease the statistical power [48, 49]. As a result, this might increase the difficulties in accurately detecting the mediating effects in a complex model. We, therefore, followed the common practice applied in previous studies that reporting the unadjusted significance level but also presenting the standard effect size as supplement [10, 14, 50]. The standard effect sizes (Cohen’s *f*^2^) of model prediction for each behavior were calculated using the formula “*f*^2^ = *R*^2^/(1-*R*^2^)”, with .02, .15, and .35 indicating a small, medium and large effect, respectively [51]. All the tests were implemented in Mplus 8 with the exemplary syntax attached in Appendix A.

## Results

### Sample characteristics

Attrition across the two data collection occasions resulted in final sample sizes of 516 participants (Mean age = 67.55 years, SD = 6.60; age range: 60- 89 years; 57.9% female; retention rate = 77.36%). Sample characteristics at follow-up are presented in the follow-up section of Table 1. Most participants were married (83.7%) and lived with their spouses, partners, or children (90.7%). Only a small percentage of participants were at primary school or below education levels (8.7%), most participants were pensioners/retired (92.6%), and more than half of the sample indicated an average level in relation to individually determined economic situation (57.9%). In addition, around half of the participants (50.8%) had suffered from chronic diseases (e.g., heart diseases, diabetes, or cancer), more than half of participants (52.7%) perceived their health status as excellent (52.7%), and only 9.7% participants reported that their family members, friends, or neighbors had been infected. Regarding BMI, more than half of sample were overweight or obese (52.1%). Attrition analyses revealed no significant differences in demographics between completers with two-wave data and dropouts without 2^nd^ wave data. However, the attrition t-tests showed that dropouts scored significantly lower in hand washing Volitional SE (t = 2.34, p = .020) and hand washing planning (t = 2.11, p = .036) than those of completers. For the rest of social cognitive variables of behaviors and three preventive behaviors, no significant differences were found.

### Findings of preliminary analyses

The means, standard deviation, internal consistency reliability (Cronbach’s α) and zero-order correlation matrices of the integrated model for three preventive behaviors are shown in Table 3. The integrated model-included variables were all significantly correlated with small-to-large effect sizes [52] except the relationship between the mask wearing behavior and the risk perception of the mask wearing (r = .067, p > .05). Particularly, the range of significant correlation coefficients was from .178 to .818 for hand washing, from .116 to .901 for mask wearing and from .170 to .896 for social distancing respectively.

**Table 3:** Means, standard deviations (SDs), reliabilities (Cronbach α), and zero-order correlation matrices of the integrated models for three preventive behaviors

**Panel 1: For hand washing behavior**													
**Variables**	**Mean**	**SD**	1	2	3	4	5	6	7	8	9	10	11
1. Motivational self-efficacy T1	93.46	15.81	.86										
2. Risk perception T1	76.34	24.36	.350^**^	.84									
3. Attitude T1	92.73	16.90	.607^**^	.395^**^	.93								
4. Subjective norm T1	95.53	13.00	.747^**^	.390^**^	.674^**^	.87							
5. Health knowledge T1	3.03	0.74	.280^**^	.221^**^	.255^**^	.390^**^	/						
6. Intention T1	95.48	13.17	.802^**^	.381^**^	.686^**^	.814^**^	.319^**^	.93					
7. Volitional self-efficacy T1	95.63	16.43	.379^**^	.178^**^	.218^**^	.219^**^	.237^**^	.351^**^	.89				
8. Planning T1	90.67	18.01	.364^**^	.240^**^	.216^**^	.198^**^	.222^**^	.349^**^	.738^**^	.87			
9. Action control T1	92.17	16.81	.357^**^	.198^**^	.254^**^	.193^**^	.228^**^	.396^**^	.748^**^	.818^**^	.91		
10. Hand washing behavior T1	3.52	0.55	.324^**^	.295^**^	.275^**^	.254^**^	.393^**^	.324^**^	.241^**^	.334^**^	.316^**^	.77	
11. Hand washing behavior T2	3.53	0.54	.238^**^	.201^**^	.189^**^	.185^**^	.311^**^	.244^**^	.371^**^	.349^**^	.337^**^	.332^**^	.77
**Panel 2: For mask wearing behavior**													
**Variables**	**Mean**	**SD**	1	2	3	4	5	6	7	8	9	10	11
1. Motivational self-efficacy T1	95.83	13.20	.98										
2. Risk perception T1	83.03	21.92	.460^**^	.92									
3. Attitude T1	93.39	16.01	.755^**^	.425^**^	.89								
4. Subjective norm T1	96.95	12.24	.855^**^	.410^**^	.671^**^	.91							
5. Health knowledge T1	3.09	0.70	.338^**^	.116^**^	.269^**^	.250^**^	/						
6. Intention T1	95.53	13.95	.861^**^	.434^**^	.721^**^	.795^**^	.283^**^	.84					
7. Volitional self-efficacy T1	94.73	14.56	.879^**^	.442^**^	.667^**^	.824^**^	.302^**^	.807^**^	.89				
8. Planning T1	93.76	15.55	.862^**^	.432^**^	.728^**^	.722^**^	.348^**^	.839^**^	.774^**^	.90			
9. Action control T1	94.82	14.25	.882^**^	.420^**^	.724^**^	.773^**^	.331^**^	.875^**^	.825^**^	.901^**^	.94		
10. Mask wearing behavior T1	3.78	0.41	.371^**^	.235^**^	.285^**^	.258^**^	.338^**^	.318^**^	.323^**^	.333^**^	.374^**^	.83	
11. Mask wearing behavior T2	3.72	0.51	.199^**^	.067	.160^**^	.161^**^	.235^**^	.211^**^	.162^**^	.222^**^	.225^**^	.322^**^	.83
**Panel 3: For social distancing behavior**													
**Variables**	**Mean**	**SD**	1	2	3	4	5	6	7	8	9	10	11
1. Motivational self-efficacy T1	94.44	15.23	.97										
2. Risk perception T1	82.59	23.18	.489^**^	.94									
3. Attitude T1	94.00	16.49	.748^**^	.441^**^	.91								
4. Subjective norm T1	95.23	14.42	.815^**^	.464^**^	.672^**^	.91							
5. Health knowledge T1	2.95	0.74	.306^**^	.147^**^	.288^**^	.296^**^	/						
6. Intention T1	94.53	15.26	.860^**^	.471^**^	.725^**^	.752^**^	.313^**^	.89					
7. Volitional self-efficacy T1	93.56	16.24	.896^**^	.491^**^	.704^**^	.752^**^	.272^**^	.841^**^	.84				
8. Planning T1	93.76	15.55	.800^**^	.441^**^	.668^**^	.665^**^	.318^**^	.770^**^	.714^**^	.91			
9. Action control T1	93.86	16.27	.890^**^	.488^**^	.739^**^	.720^**^	.318^**^	.829^**^	.855^**^	.786^**^	.95		
10. Social distancing behavior T1	3.69	0.46	.303^**^	.213^**^	.216^**^	.211^**^	.355^**^	.264^**^	.264^**^	.301^**^	.302^**^	.87	
11. Social distancing behavior T2	3.61	0.47	.342^**^	.170^**^	.212^**^	.240^**^	.323^**^	.302^**^	.292^**^	.309^**^	.333^**^	.295^**^	.87

Note. T1=Time 1. T2=Time 2.

^**^ *p* < .01. Correlation coefficient (r) values below 0.3 are small, 0.3-0.7 are moderate, >0.7 are large (Cohen, 1992).

Internal consistency reliability (Cronbach α) is displayed on the diagonal line. As there is only one item for health knowledge of each preventive behavior, reliability of health knowledge is not available and presented as “/”.

### Findings of SEM

According to the model fit and quality indices, the overall fit indices of the proposed models regarding all three preventive behaviors (both including and excluding past behaviors) are acceptable (see Table 4 for details).

**Table 4:** Fit indices of the Integrated Social Cognition Model regarding all three preventive behaviors with and without past behaviors

Model of Behavior	Degree of freedom	Chi-square	CFI	TLI	SRMR	RMSEA	RMSEA 90% CI
1. Hand washing	14	42.80^***^	.991	.978	.021	.060	.039 - .081
2. Hand washing (past behavior included)	14	39.57^***^	.992	.978	.017	.056	.035 - .078
3. Mask wearing	14	40.18^***^	.991	.980	.014	.062	.042 - .084
4. Mask wearing (past behavior included)	14	45.77^***^	.991	.977	.013	.063	.043 - .084
5. Social distancing	14	39.12^***^	.992	.981	.015	.059	.038 - .081
6. Social distancing (past behavior included)	14	32.88^**^	.994	.983	.012	.051	.028 - .074

*CFI* Comparative Fit Index, *TLI* Tucker-Lewis Index, *SRMR* Standardized Root Mean Square Residual, *RMSEA* Root Mean Square Error of Approximation, *CI* Confidence Interval

For CFI and TLI, values ≥.90 indicating acceptable model fit, values ≥.95 indicating good model fit; For SRMR and RMSEA, values with .08 or less indicating acceptable model fit, values with .05 or less indicating good model fit. ** *p* < .01; *** *p* < .001

**Fig. 2:**
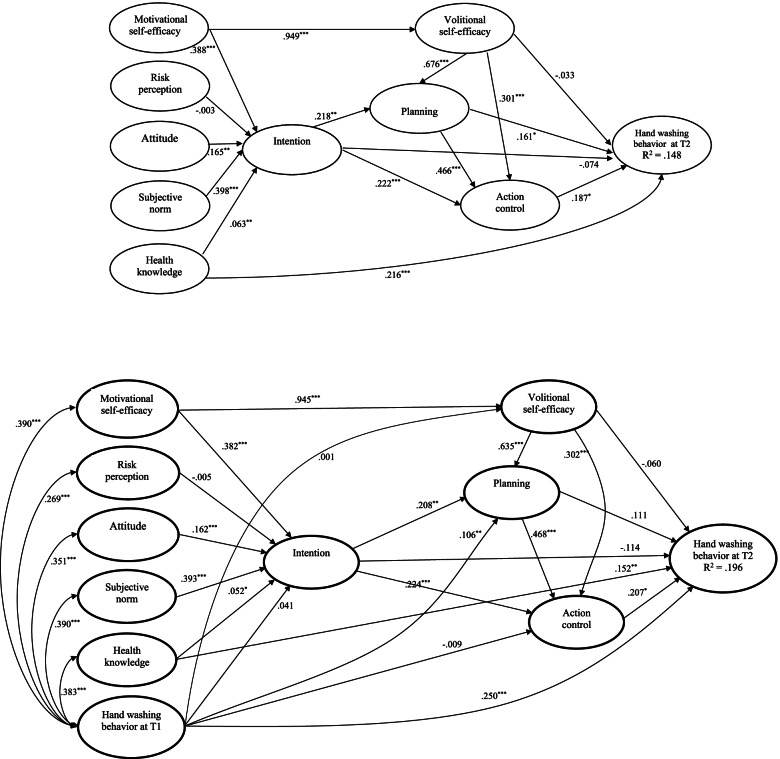
Standardized parameter estimates of the Integrated Social Cognition Model regarding hand washing behavior. Note. The upper panel presents the model excluding past behavior and the lower panel presents the model including past behavior. * *p* < .05; ** *p* < .01; *** *p* < .001

For hand washing, standardized parameter estimates for the hypothesized structural relations of the integrated model excluding past behavior are presented in Fig. 2. The social cognition constructs explained 14.8% variance of hand washing with a medium effect size (*R*^2^ = .148, *f*^2^ = .174). For the direct effects, significant relationships appeared in 13 out of 16 hypothesized associations (Motivational SE to intention, β = .388, *p* < .001; attitude to intention, β = .165, *p* < .001; subjective norm to intention, β = .398, p < .001; health knowledge to intention, β = .063, *p* = .004; Motivational SE to Volitional SE, β = .949, *p* < .001; intention to planning, β = .218, *p* = .003; Volitional SE to planning, β = .676, *p* < .001; intention to action control, β = .222, *p* < .001; planning to action control, β = .466, *p* < .001; Volitional SE to action control, β = .301, *p* < .001; health knowledge to hand washing, β = .216, *p* < .001; planning to hand washing, β = .161, *p*= .035; action control to hand washing, β = .187, *p* = .043). Furthermore, subjective norm was the strongest predictor of intention compared to Motivational SE, attitude, and health knowledge. Volitional SE was the strongest predictor of planning compared to intention. Planning was the strongest predictor of action control compared to intention and Volitional SE. Health knowledge was the strongest predictor of hand washing compared to planning and action control. In addition, only three hypothesized relationships were nonsignificant (risk perception to intention, β = -.003, *p* = .890; intention to hand washing, β = -.074, *p* = .352; Volitional SE to hand washing, β = -.033, *p* = .653). For the indirect effects, 3 out of 11 hypothesized mediating relationships were supported. Specifically, planning significantly mediated between Volitional SE and hand washing (β = .109, *p* = .042), action control significantly mediated between Volitional SE and hand washing (β = .056, *p* = .047) as well as between planning and hand washing (β = .087, *p* = .044) respectively. In addition, action control showed a mediating role at the marginal effects between intention and hand washing (β = .042, p = .054).

When past behavior of hand washing was included in the model, significant effects of past behavior on all model constructs and hand washing at T2 were observed except on intention (β = .041, *p* = .096), Volitional SE (β = .001,***p*** = .960) and action control (β = -.009, *p* = .687) (see Fig. 2). Inclusion of past behavior in the model increased the variance explained in hand washing behavior with a medium effect size (*R*^2^ = .196, *f*^2^ = .24) but led to an attenuation of the structural relations. Specifically, the effect of planning on behavior became nonsignificant (β = .111, *p* = .141). In addition, among another 12 significant effects, eight reduced (Motivational SE to Volitional SE, Motivational SE to intention, attitude to intention, subjective norm to intention, health knowledge to intention, intention to planning, health knowledge to hand washing, Volitional SE to planning) and four slightly increased (see Fig. 2). A full breakdown of estimates on the integrated model of hand washing with and without past behavior, including direct, indirect and total effects is presented in supplementary Appendix B.

**Fig. 3:**
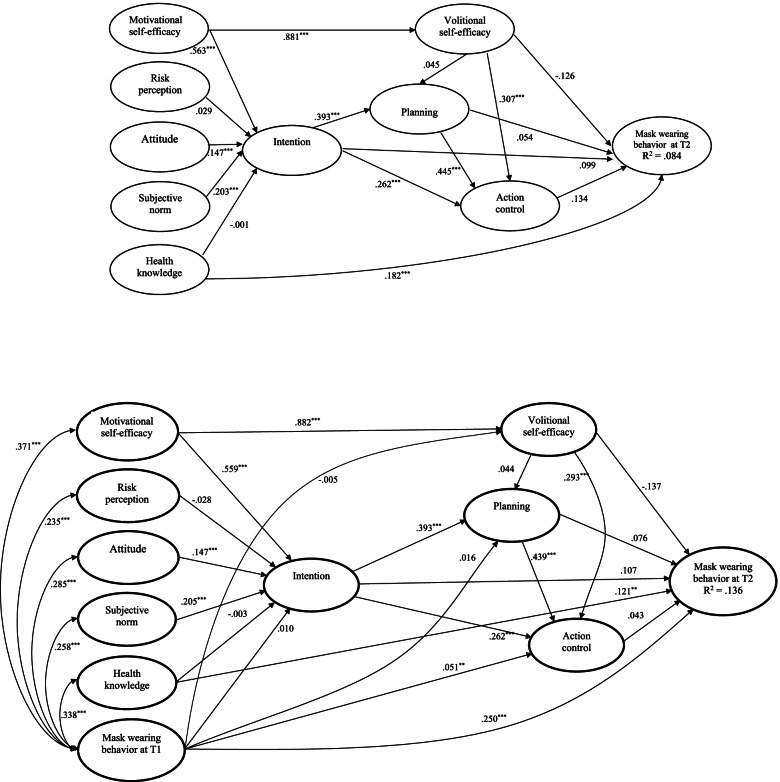
Standardized parameter estimates of the Integrated Social Cognition Model regarding mask wearing behavior. Note. The upper panel presents the model excluding past behavior and the lower panel presents the model including past behavior. * p < .05; ** p < .01; *** p < .001

For mask wearing, standardized parameter estimates for the hypothesized structural relations of the integrated model excluding past behavior are presented in Fig. 3. The social cognition constructs explained 8.4% variance of mask wearing with a small effect size (*R*^2^ = .084*, f*^2^ = .092). For the direct effects, significant relationships appeared in 9 out of 16 hypothesized associations (Motivational SE to intention, β = .563, p < .001; attitude to intention, β = .147, p < .001; subjective norm to intention, β = .203, p < .001; Motivational SE to Volitional SE, β = .881, p < .001; intention to planning, β = .393 , *p* < .001; intention to action control, β = .262, p < .001; planning to action control, β = .445, *p* < .001; Volitional SE to action control, β = .307, p < .001; health knowledge to mask wearing, β = .182, *p* < .001). Furthermore, Motivational SE was the strongest predictor of intention compared to attitude and subjective norm. Intention was the only predictor of planning. Planning was the strongest predictor of action control compared to Volitional SE and intention. Health knowledge was the only predictor of mask wearing. In addition, seven hypothesized relationships were nonsignificant (risk perception to intention, β = .029, *p* = .232; health knowledge to intention, β = -.001, *p* = .972; Volitional SE to planning, β = .045, p = .308; intention to mask wearing, β = .099, *p* = .287; planning to mask wearing, β = .054, *p* = .596; Action control to Mask Wearing, β = .134, p = .252; Volitional SE to mask wearing, β = -.126, p = .109). For the indirect effects, none of the hypothesized mediating relationships for mask wearing were supported.

When past behavior of mask wearing was included in the model, significant effects of past behavior on all model constructs and hand washing at T2 were observed except on intention (β = .010, p = .669), Volitional SE (β = -.005, p = .837) and planning (β = -.016, p = .471) (see Fig. 3). Inclusion of past behavior in the model increased the variance explained in mask wearing behavior with a medium effect size (*R*^2^ = .136, *f*^2^ = .157) but led to an attenuation of the structural relations. Specifically, among nine significant effects, four reduced (Motivational SE to intention, health knowledge to behavior, Volitional SE to action control, planning to action control), three unchanged (attitude to intention; intention to planning, and intention to action control) and two slightly increased (Motivational SE to Volitional SE, subjective norm to intention) (see Fig. 3). A full breakdown of estimates on the integrated model of mask wearing with and without past behavior, including direct, indirect and total effects is presented in supplementary Appendix C.

**Fig. 4:**
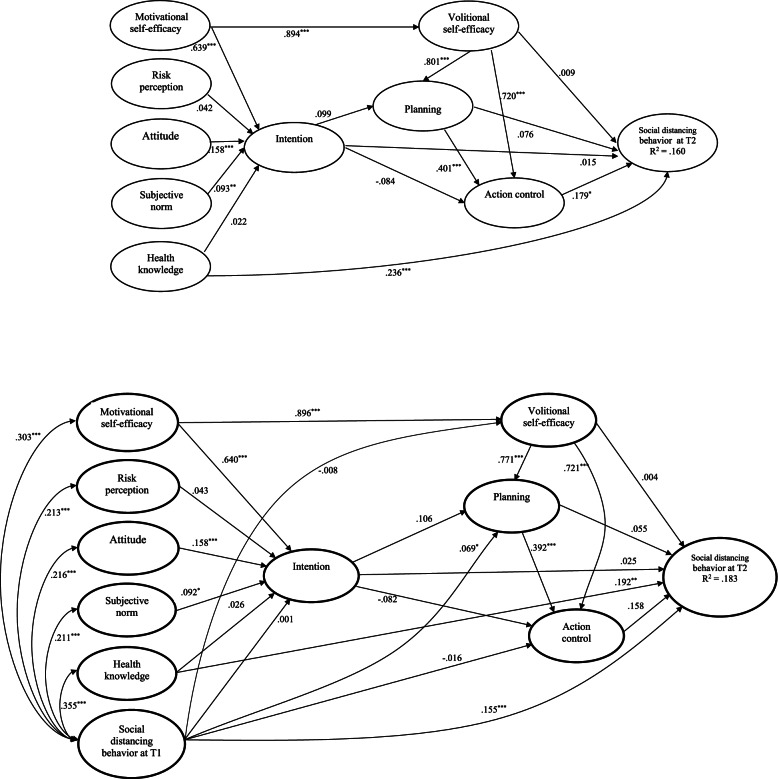
Standardized parameter estimates of the Integrated Social Cognition Model regarding social distancing behavior. Note. The upper panel presents the model excluding past behavior and the lower panel presents the model including past behavior. * p < .05; ** p < .01; *** p < .001

For social distancing, standardized parameter estimates for the hypothesized structural relations of the integrated model excluding past behavior are presented in Fig. 4. The social cognition constructs explained 16% variance of social distancing with a medium effect size (*R*^2^ = .160, *f*^2^ = .190). For the direct effects, significant relationships appeared in 9 out of 16 hypothesized associations (Motivational SE to intention, β = .639, *p* < .001; Attitude to Intention, β = .158, *p* < .001; subjective norm to intention, β = .093, p = .009; Motivational SE to Volitional SE, β = .894, *p* < .001; Volitional SE to planning, β = .801, *p* < .001; planning to action control, β = .401, *p* < .001; Volitional SE to action control, β = .720, *p* < .001; health knowledge to social distancing, β = .236, p < .001; action control to social distancing, β = .179, p = .049). Furthermore, Motivational SE was the strongest predictor of intention compared to attitude and subjective norm. Volitional SE was the only predictor of planning. Volitional SE was the strongest predictor of action control compared to planning. Health knowledge was the strongest predictor of social distancing compared to action control. In addition, seven hypothesized relationships were nonsignificant (health knowledge to intention, β = .022, p = .304; risk perception to intention, β = .042, p = .091; intention to planning, β = .099, p = .214; intention to action control, β = -.084, p = .136; planning to social distancing, β = .076, p = .278; Volitional SE to social distancing, β = .009, p = .915; intention to social distancing, β = .015, p = .861). For the indirect effects, none of the hypothesized mediating relationships were supported. Notably, there were marginally significant indirect effects of Volitional SE (β = .13, p = .051) and planning (β = .08, p = .053) on social distancing mediated by action control.

When past behavior of social distancing was included in the model, significant effects of past behavior on all model constructs and social distancing at T2 were observed except on intention (β = -.001, p = .964), Volitional SE (β =- .008, *p* = .710) and action control (β = .016, p = .481) (see Fig. 4). Inclusion of past behavior in the model increased the variance explained in social distancing behavior with a medium effect size (*R*^2^ =.183, *f*^2^ = .224) but led to an attenuation of the structural relations. Specifically, the effect of action control on behavior became nonsignificant (β = .158, *p* = .079). In addition, among the other eight significant effects, four reduced (subjective norm to intention, Volitional SE to planning, planning to action control, health knowledge to social distancing), one unchanged (attitude to intention) and three slightly increased (Motivational SE to Volitional SE, Motivational SE to intention, Volitional SE to action control) (see Fig. 4). A full breakdown of estimates on the integrated model of social distancing with and without past behavior, including direct, indirect and total effects is presented in supplementary Appendix D.

## Discussion

### Main findings

This two-wave prospective study aimed to identify the determinants of three preventive behaviors (hand washing, mask wearing, and social distancing) of older adults in the context of COVID-19, through the application of an integrated social cognition model. Results indicated that the hypothesized model generally had acceptable predictability regarding all three preventive behaviors. Among the 16 hypothesized direct paths for each preventive behavior, seven paths (attitude, Motivational SE and subjective norm to intention respectively; Motivational SE to Volitional SE; planning and Volitional SE to action control respectively; health knowledge to behavior) were significant in models for all three preventive behaviors. While three paths (risk perception to intention; intention to behavior; and Volitional SE to behavior) were not significant in any of the three models. The other six paths were statistically significant in one or two models of preventive behaviors. The findings partially supported hypothesis 1. For the mediation effects, action control was revealed as a mediator for the relationship between Volitional SE and hand washing, as well as for the relationship between planning and hand washing. Such mediating roles of action control also appeared in the model of social distancing behavior but with marginal effects. The findings partially supported hypothesis 2. In addition, all three preventive behaviors could be significantly predicted by past behaviors. The inclusion of past behaviors in three models attenuated most of the structural relations, which partially supported hypothesis 3.

Notably, paths of motivational self-efficacy and subjective norm to intention, volitional self-efficacy to planning, planning to action control had medium to strong relationships in all three models. These findings were partially consistent with recent studies on the preventive behaviors in other age groups [53]. Paths directly from health knowledge to all three preventive behaviors appear to be significant. Such findings can also be referred in previous studies among adults and adolescents [54–56]. The results emphasized the importance of health knowledge about how and in what situations to enact preventive behaviors when facilitating older adults to perform preventive behaviors. In contrast to earlier findings [10, 14], no significant direct associations from intention to each of preventive behaviors were detected in this study. This may be because the measurements of intention are different among studies.

When focusing on hand washing behavior, it was found that health knowledge, planning and action control was directly associated with hand washing behavior. Besides, it was also shown that action control served as mediators between planning and hand washing as well as between volitional self-efficacy and hand washing. However, the intention-involved direct and indirect relationships to hand washing behavior were all nonsignificant. The results above imply the importance of health knowledge education in facilitating hand washing behavior among older adults. In addition, such behavior enactment required more self-regulatory process (accurate planning and rigorous action control) than merely sufficient intention. Previous studies also highlight the importance of self-regulation on behavior enactment and maintenance in the context of COVID-19 prevention [57], thus future interventions might focus more on educating older adults how to appropriately make plans and monitor themselves in complying with hand washing behavior during the COVID-19 pandemic.

Regarding mask wearing behavior, there was only one direct significant path from health knowledge to behavior. Such a direct relationship is consistent with other studies and suggests that knowledge instruction is very useful in promoting mask wearing behavior under pandemic situations [54, 55].

As to social distancing behavior, it was found that health knowledge was significantly associated with social distancing behavior. This relationship agrees with a recent study on British young adults [58]. Importantly, action control also showed direct relationships with social distancing whereas the association disappeared with the inclusion of past behavior. The finding implies that future interventions need to educate older adults how to keep constant awareness on keeping social distancing. For those who do not have stable behavioral habits, it is still crucial to take efforts and self-monitor social distancing in public areas (e.g., staying out of crowded places or mass gatherings when in public, maintaining at least 1-meter distance between self and other people who were coughing or sneezing) [31].

It is worth noting that the three preventive behaviors differed prominently in the relationship with several modifiable factors in our study. For example, action control only predicted handwashing but not facemask wearing and social distancing. The potential reason for this might be that the enactment of preventive behaviors is influenced by both internal (e.g., self-efficacy, planning, self-monitoring) and external sources (e.g., cue-to-action, policy and social environment) [25, 59, 60]. For facemask wearing and social distancing which have to be performed compulsorily in the public areas in China [61], the external sources (i.e., government policy) might outweigh the internal sources (i.e., action control), thereby suppress the prediction of internal sources for the behavioral execution. Nevertheless, this assumption has not been systematically examined in this study and deserves further research.

The unique findings of the current paper imply that more community-based workshops and campaigns can be organized to enrich the health knowledge of three preventive behaviors in older adults. In addition, several behavioral change techniques (e.g., self-monitoring, planning) can be applied to enhance the maintenance of handwashing behavior [62]. This is also necessary for facemask wearing and social distancing especially now the compulsory policy has been released. Strategies to promote the formation of behavioral habits (e.g., adding more social cues) should also be considered in future interventions promoting preventive behaviors in older adults [63].

### Strengths and limitations

To the best of our knowledge, this is the first prospective study using an online survey to identify the determinants of all three individual preventive behaviors among older adults during the COVID-19 pandemic. There are two major strengths of the present study. The first strength relates to study design and methodology. We adopted the prospective design with a relatively large sample size. By using the statistical method of SEM, the social-cognition constructs of the preventive behavior of COVID-19 were revealed. The second strength lies to the rareness of our sample. The sample of older adults was extremely difficult to reach using an online survey. We investigated Chinese older adults in Hubei Province, which was the most severe region during the COVID-19 outbreak in China during implementation of our research. By exploiting the personal relationship of all the authors and obtaining the support of officers from the local communities, first-hand data was collected. We believe that our study can offer valuable understanding and explanation of COVID-19 preventive behaviors among older adults. The research findings can inform interventions which aim at promoting the enactment of older adults’ preventive behaviors during the COVID-19 and future pandemics.

The limitations of the current study should also be mentioned. First, since the sampling was not based on a random approach, the representativeness of participants is questionable. Second, all the variables were measured by self-reported scales which might lead to recall bias and social desirability effects. Third, although it is most efficient and cost-effective to conduct the survey via an online platform under these special circumstances and while several strategies were adopted to ensure the retention rate, there was still a 22.6% participant drop out at T2. Fourth, it should be noted that the model explanations on variance of preventive behaviors were relatively low (8.4%-16% excluding past behaviors; 13.6%-18.3% including past behaviors). More social cognition constructs such as habit need to be explored in future research among older adults [10]. Fifth, the adjusted approach was not used in the current study to control for the Type-I error when reporting correlations and different model paths. This issue should be addressed and deserves further discussion in the future [64, 65]. Finally, the influence of demographic variables on the predictive model for three types of preventive behaviors was not examined in the current study. Future research should explore this area further.

### Conclusions

The current study identified key modifiable determinants of three preventive behaviors in a sample of Chinese older adults based on integrated social cognition models during the COVID-19 pandemic. The findings provide acceptable support for the proposed model, highlighting the importance of social cognitive constructs such as health knowledge and action control in predicting behavior. Findings suggest that future interventions should enhance health knowledge and strengthen the capability of action control in facilitating preventive behaviors among older adults.

## Supplementary Information

**Additional file 1.** Appendices

## Data Availability

Requests of data and materials should be directed to the study director: Dr. Duan Yanping (duanyp@hkbu.edu.hk).

## References

[1] Statement – Older people are at highest risk from COVID-19, but all must act to prevent community spread [Internet]. euro.who.int. [cited 2021 Sep 9]. Available from: https://www.euro.who.int/en/health-topics/health-emergencies/coronavirus-covid-19/statements/statement-older-people-are-at-highest-risk-from-covid-19,-but-all-must-act-to-prevent-community-spread.

[2] Shahid Z, Kalayanamitra R, McClafferty B, Kepko D, Ramgobin D, Patel R (2020). COVID-19 and older adults: What we know: COVID-19 in older adults. J Am Geriatr Soc.

[3] US Centers for Disease Control and Prevention. How to protect yourself & others [Internet]. Cdc.gov 2021 [cited 2021 Sep 9]. Available from: https://www.cdc.gov/coronavirus/2019-ncov/prevent-getting-sick/prevention.html

[4] Advice for the public on COVID-19 – World Health Organization [Internet]. Who.int. [cited 2021 Sep 9]. Available from: https://www.who.int/emergencies/diseases/novel-coronavirus-2019/advice-for-public?gclid=Cj0KCQjwg7KJBhDyARIsAHrAXaFlpfhsQreq3Yws7As7eWeHXXcYX3UUS6mrohDoGvSN-130LtcYpP8aAnNCEALw_wcB

[5] Promoting mask-wearing during the COVID-19 pandemic: A policymaker’s guide [Internet]. Africacdc.org. 2020 [cited 2021 Sep 9]. Available from: https://africacdc.org/download/promoting-mask-wearing-during-the-covid-19-pandemic-a-policymakers-guide/

[6] Bonell C, Michie S, Reicher S (2020). Harnessing behavioural science in public health campaigns to maintain “social distancing” in response to the COVID-19 pandemic: Key principles. J Epidemiol Commun Health.

[7] Chen Y, Zhou R, Chen B, Chen H, Li Y, Chen Z, et al. Knowledge, perceived beliefs, and preventive behaviors related to COVID-19 among Chinese older adults: Cross-sectional web-based survey (preprint) [Internet]. JMIR Preprints 2020. Available from: 10.2196/preprints.23729 10.2196/23729 PMC7781588 33293262

[8] Haischer MH, Beilfuss R, Hart MR, Opielinski L, Wrucke D, Zirgaitis G (2020). Who is wearing a mask? Gender-, age-, and location-related differences during the COVID-19 pandemic. PLoS Ones.

[9] Raude J, Lecrique J-M, Lasbeur L, Leon C, Guignard R, du Roscoät E (2020). Determinants of preventive behaviors in response to the COVID-19 pandemic in France: Comparing the sociocultural, psychosocial, and social cognitive explanations. Front Psychol.

[10] Hagger MS, Smith SR, Keech JJ, Moyers SA, Hamilton K (2020). Predicting social distancing intention and behavior during the COVID-19 pandemic: An integrated social cognition model. Ann Behav Med.

[11] Derksen C, Keller FM, Lippke S (2020). Obstetric healthcare workers’ adherence to hand hygiene recommendations during the COVID-19 pandemic: Observations and social-cognitive determinants. Appl Psychol Health Well Being.

[12] Ajzen I (1985). From intentions to actions: A theory of planned behavior. Action Control.

[13] Gaube S, Fischer P, Lermer E (2021). Hand (y) hygiene insights: Applying three theoretical models to investigate hospital patients’ and visitors’ hand hygiene behavior. PloS one.

[14] Zhang C-Q, Fang R, Zhang R, Hagger MS, Hamilton K (2020). Predicting hand washing and sleep hygiene behaviors among college students: Test of an integrated social-cognition model. Int J Environ Res Public Health.

[15] Chung P-K, Zhang C-Q, Liu J-D, Chan DK-C, Si G, Hagger MS (2017). The process by which perceived autonomy support predicts motivation, intention, and behavior for seasonal influenza prevention in Hong Kong older adults. BMC Public Health.

[16] Sniehotta FF, Presseau J, Araújo-Soares V (2014). Time to retire the theory of planned behaviour. Health Psychol Rev.

[17] McEachan RRC, Conner M, Taylor NJ, Lawton RJ (2011). Prospective prediction of health-related behaviours with the Theory of Planned Behaviour: a meta-analysis. Health Psychol Rev.

[18] Hagger MS (2015). Retired or not, the theory of planned behaviour will always be with us. Health Psychol Rev.

[19] Dumitrescu AL, Wagle M, Dogaru BC, Manolescu B (2011). Modeling the theory of planned behavior for intention to improve oral health behaviors: the impact of attitudes, knowledge, and current behavior. J Oral Sci.

[20] Tao SY, Cheng YL, Lu Y, Hu YH, Chen DF (2013). Handwashing behaviour among Chinese adults: a cross-sectional study in five provinces. Public Health.

[21] Ajilore K, Atakiti I, Onyenankeya K (2017). College students’ knowledge, attitudes and adherence to public service announcements on Ebola in Nigeria: Suggestions for improving future Ebola prevention education programmes. Health Educ J.

[22] Zhong B-L, Luo W, Li H-M, Zhang Q-Q, Liu X-G, Li W-T (2020). Knowledge, attitudes, and practices towards COVID-19 among Chinese residents during the rapid rise period of the COVID-19 outbreak: a quick online cross-sectional survey. Int J Biol Sci.

[23] Chen X, Ran L, Liu Q, Hu Q, Du X, Tan X (2020). Hand hygiene, mask-wearing behaviors and its associated factors during the COVID-19 epidemic: A cross-sectional study among primary school students in Wuhan, China. Int J Environ Res Public Health.

[24] Vicerra PMM (2021). Knowledge-behavior gap on COVID-19 among older people in rural Thailand. Gerontol Geriatr Med.

[25] Schwarzer R (2008). Modeling health behavior change: How to predict and modify the adoption and maintenance of health behaviors. Appl Psychol.

[26] Lao CK, Li X, Zhao N, Gou M, Zhou G. Using the health action process approach to predict facemask use and hand washing in the early stages of the COVID-19 pandemic in China. Curr Psychol. 2021:1-10. 10.1007/s12144-021-01985-0. 10.1007/s12144-021-01985-0 PMC8210514 34155429

[27] Hamilton K, Smith SR, Keech JJ, Moyers SA, Hagger MS (2020). Application of the health action process approach to social distancing behavior during COVID-19. Appl Psychol Health Well Being.

[28] Sobkow A, Zaleskiewicz T, Petrova D, Garcia-Retamero R, Traczyk J (2020). Worry, risk perception, and controllability predict intentions toward COVID-19 preventive behaviors. Front Psychol.

[29] Sutton S (2008). How does the health action process approach (HAPA) bridge the intention–behavior gap? An examination of the model’s causal structure. Appl Psychol.

[30] Hagger MS, Luszczynska A (2014). Implementation intention and action planning interventions in health contexts: state of the research and proposals for the way forward: Planning interventions: The way forward. Appl Psychol Health Well Being.

[31] Sniehotta FF, Nagy G, Scholz U, Schwarzer R (2006). The role of action control in implementing intentions during the first weeks of behaviour change. Br J Soc Psychol..

[32] Chow S, Mullan B (2010). Predicting food hygiene. An investigation of social factors and past behaviour in an extended model of the Health Action Process Approach. Appetite.

[33] Reyes Fernández B, Knoll N, Hamilton K, Schwarzer R (2016). Social-cognitive antecedents of hand washing: Action control bridges the planning-behaviour gap. Psychol Health.

[34] Pinidiyapathirage J, Jayasuriya R, Cheung NW, Schwarzer R (2018). Self-efficacy and planning strategies can improve physical activity levels in women with a recent history of gestational diabetes mellitus. Psychol Health.

[35] Schwarzer R, Antoniuk A, Gholami M (2015). A brief intervention changing oral self-care, self-efficacy, and self-monitoring. Br J Health Psychol.

[36] Reyes Fernández B, Fleig L, Godinho CA, Montenegro Montenegro E, Knoll N, Schwarzer R (2015). Action control bridges the planning-behaviour gap: a longitudinal study on physical exercise in young adults. Psychol Health.

[37] Luszczynska A, Schwarzer R. Social cognitive theory. Fac Health Sci Publ. 2015:225–51.

[38] Liang W, Duan Y, Shang B, Hu C, Baker JS, Lin Z (2021). Precautionary behavior and depression in older adults during the COVID-19 pandemic: An online cross-sectional study in Hubei, China. Int J Environ Res Public Health.

[39] Duan YP, Wienert J, Hu C, Si GY, Lippke S (2017). Web-based intervention for physical activity and fruit and vegetable intake among Chinese university students: A randomized controlled trial. J Med Internet Res.

[40] Li X, Liu Q (2020). Social media use, eHealth literacy, disease knowledge, and preventive behaviors in the COVID-19 pandemic: Cross-sectional study on Chinese netizens. J Med Internet Res.

[41] Rosen L, Zucker D, Brody D, Engelhard D, Manor O (2009). The effect of a handwashing intervention on preschool educator beliefs, attitudes, knowledge and self-efficacy. Health Educ Res.

[42] Duan YP, Liang W, Guo L, Wienert J, Si GY, Lippke S (2018). Evaluation of a Web-based intervention for multiple health behavior changes in patients with coronary heart disease in home-based rehabilitation: Pilot randomized controlled trial. J Med Internet Res.

[43] Liang W, Duan YP, Shang BR, Wang YP, Hu C, Lippke S (2019). A web-based lifestyle intervention program for Chinese college students: study protocol and baseline characteristics of a randomized placebo-controlled trial. BMC Public Health.

[44] Little RJA, Rubin DB (1989). The analysis of social science data with missing values. Sociol Methods Res.

[45] Kim H-Y (2013). Statistical notes for clinical researchers: assessing normal distribution (2) using skewness and kurtosis. Restor Dent Endod.

[46] Shah RB (2012). A multivariate analysis technique: Structural equation modeling. Asian Journal of Multidimensional. Research.

[47] Weber R (2007). Responses to matsunaga: To adjust or not to adjust alpha in multiple testing: That is the question. Guidelines for alpha adjustment as response to O’Keefe’s and matsunaga’s critiques. Commun Methods Meas.

[48] Althouse AD (2016). Adjust for multiple comparisons? It’s not that simple. Ann Thoracic Surg.

[49] Mudge JF, Baker LF, Edge CB, Houlahan JE (2012). Setting an optimal α that minimizes errors in null hypothesis significance tests. PloS One.

[50] Hagger MS, Chatzisarantis NL (2014). An integrated behavior change model for physical activity. Exerc Sport Sci Rev.

[51] Selya AS, Rose JS, Dierker LC (2012). A practical guide to calculating Cohen’s f2, a measure of local effect size, from PROC MIXED. Front Psychol.

[52] Cohen J. A power primer. Psychological bulletin.1992;112(1): 155. 10.1037//0033-2909.112.1.155 19565683

[53] Alivernini F, Manganelli S, Girelli L, Cozzolino M, Lucidi F, Cavicchiolo E (2021). Physical distancing behavior: The role of emotions, personality, motivations, and moral decision-making. J Pediatr Psychol.

[54] Cui T, Yang G, Ji L, Zhu L, Zhen S, Shi N (2020). Chinese residents’ perceptions of COVID-19 during the pandemic: Online cross-sectional survey study. J Med Internet Res.

[55] Dardas LA, Khalaf I, Nabolsi M, Nassar O, Halasa S (2020). Developing an understanding of adolescents’ knowledge, attitudes, and practices toward COVID-19. J Sch Nurs.

[56] Desalegn Z, Deyessa N, Teka B, Shiferaw W, Hailemariam D, Addissie A (2021). COVID-19 and the public response: Knowledge, attitude and practice of the public in mitigating the pandemic in Addis Ababa, Ethiopia. PLoS One..

[57] Pacholik-Żuromska A (2021). Self-regulation in the time of lockdown. Front Neuroinform.

[58] Barrett C, Cheung KL (2021). Knowledge, socio-cognitive perceptions and the practice of hand hygiene and social distancing during the COVID-19 pandemic: a cross-sectional study of UK university students. BMC Public Health.

[59] Abraham C, Sheeran P, Johnston M (1998). From health beliefs to self-regulation: Theoretical advances in the psychology of action control. Psychol Health.

[60] Fitzsimons G M, Bargh J A. Automatic self-regulation. Handbook of self-regulation: Research, theory, and applications.2004;151-170.

[61] Epidemic prevention and control _ China Government Network [Internet]. Gov.cn. [cited 2021 Sep 9]. Available from: http://www.gov.cn/fuwu/zt/yqfkzq/index.htm

[62] Michie S, West R, Sheals K, Godinho CA (2018). Evaluating the effectiveness of behavior change techniques in health-related behavior: a scoping review of methods used. Transl Behav Med.

[63] Hagger MS. Redefining habits and linking habits with other implicit processes. 2020;46(101606):Psychol Sport Exerc, 101606.

[64] Green SB, Babyak MA (1997). Control of Type I errors with multiple tests of constraints in structural equation modeling. Multivariate Behav Ress.

[65] Smith, C. E., & Cribbie, R. A. (2013). Multiplicity control in structural equation modeling: Incorporating parameter dependencies. Structural Equation Modeling: A Multidisciplinary Journal, 20(1), 79-85.

