# Supplementary material for: Predicting hand washing, mask wearing and social distancing behaviors among older adults during the covid-19 pandemic: an integrated social cognition model
Source: BMC Geriatr. 2022 Feb 2;22:91. doi: 10.1186/s12877-022-02785-2 (PMC8807958; doi:10.1186/s12877-022-02785-2)
Supplement: Supplementary file 1 — Additional file 1. Appendices [file 12877_2022_2785_MOESM1_ESM.docx]

Appendix A:

Mplus VERSION 8 MUTHEN & MUTHEN 02/27/2021 3:44 PM

INPUT INSTRUCTIONS TITLE:

HW excluding previous DATA:

FILE IS F:\Publication\dataset for mplus\New folder\HW.DAT; VARIABLE:

NAMES ARE PRE PSE RK ATT SN KL INT VSE PL AC POST; USEVARIABLES ARE PSE RK ATT SN KL INT VSE PL AC POST; ANALYSIS:

TYPE = GENERAL; ESTIMATOR = ML; OUTPUT:

SAMPSTAT STANDARDIZED RESIDUAL CINTERVAL MODINDICES (10.0) TECH2 TECH4; MODEL:

INT ON PSE RK SN KL ATT; VSE ON PSE;

PL ON INT VSE; AC ON INT PL VSE;

POST ON KL INT VSE PL AC; PSE WITH RK ATT SN KL; RK WITH ATT SN KL;

ATT WITH SN KL; SN WITH KL;

PL WITH VSE; VSE WITH INT; PSE WITH VSE; KL WITH PL;

MODEL INDIRECT: POST IND INT; POST IND VSE; POST IND PL;

*Appendix B*

*Standardized Parameter Estimates for the Direct, Indirect, and Total Effects in the Integrated Model of Hand Washing Behavior Excluding and Including Past Behavior*

**Effects Model excluding past behavior Model including past behavior β *p* 95% CI β *p* 95% CI**

**LB UB LB UB**

**Direct Effects**

MSE→INT .388 <.001 .325 .452 .382 <.001 .319 .446

| RP→INT | -.003 | .890 | -.048 | .042 | -.005 | .816 | -.050 | .040 |
| --- | --- | --- | --- | --- | --- | --- | --- | --- |
| ATT→INT | .165 | <.001 | .108 | .222 | .162 | <.001 | .106 | .219 |
| SN→INT | .398 | <.001 | .331 | .465 | .393 | <.001 | .325 | .460 |
| HK→INT | .063 | .004 | .020 | .105 | .052 | .022 | .008 | .096 |
| HK→HW | .216 | <.001 | .131 | .301 | .152 | .001 | .066 | .239 |
| MSE→VSE | .949 | <.001 | .913 | .985 | .945 | <.001 | .896 | .994 |
| INT→PL | .218 | .003 | .072 | .364 | .208 | .004 | .066 | .351 |
| INT→AC | .222 | <.001 | .151 | .294 | .224 | <.001 | .152 | .296 |
| INT→HW | -.074 | .352 | -.229 | .082 | -.114 | .141 | -.265 | .038 |
| VSE→PL | .676 | <.001 | .515 | .836 | .635 | <.001 | .477 | .792 |
| VSE→AC | .301 | <.001 | .239 | .363 | .302 | <.001 | .239 | .364 |
| VSE→HW | -.033 | .653 | -.175 | .109 | -.060 | .393 | -.198 | .078 |
| PL→AC | .466 | .000 | .408 | .523 | .468 | <.001 | .409 | .527 |
| PL→HW | .161 | .035 | .011 | .312 | .111 | .141 | -.037 | .259 |
| AC→HW | .187 | .043 | .006 | .368 | .207 | .021 | .031 | .382 |
| PB ∝ MSE | / | / | / | / | .390 | <.001 | .317 | .463 |
| PB ∝ RP | / | / | / | / | .269 | <.001 | .189 | .349 |
| PB ∝ ATT | / | / | / | / | .351 | <.001 | .275 | .427 |
| PB ∝ SN | / | / | / | / | .390 | <.001 | .317 | .464 |
| PB ∝ HK | / | / | / | / | .383 | <.001 | .309 | .457 |
| PB→INT | / | / | / | / | .041 | .096 | -.007 | .089 |
| PB→PL | / | / | / | / | .106 | .002 | .040 | .173 |
| PB→VSE | / | / | / | / | .001 | .960 | -.051 | .054 |
| PB→AC | / | / | / | / | -.009 | .687 | -.051 | .034 |
| PB→HW | / | / | / | / | .250 | <.001 | .162 | .338 |
| **Indirect Effects**  MSE→INT→HW | -.029 | .354 | -.089 | .032 | -.044 | .144 | -.102 | .015 |
| RP→INT→HW | .000 | .891 | -.003 | .004 | .001 | .818 | -.005 | .006 |

| ATT→INT→HW | -.012 | .359 | -.038 | .014 | -.018 | .154 | -.044 | .007 |
| --- | --- | --- | --- | --- | --- | --- | --- | --- |
| SN→INT→HW | -.029 | .354 | -.091 | .033 | -.045 | .144 | -.105 | .015 |
| HK→INT→HW | -.005 | .376 | -.015 | .006 | -.006 | .215 | -.015 | .003 |
| MSE→VSE→HW | -.031 | .653 | -.166 | .104 | -.057 | .394 | -.188 | .074 |
| INT→PL→HW | .035 | .088 | -.005 | .076 | .023 | .191 | -.012 | .058 |
| INT→AC→HW | .042 | .054 | -.001 | .084 | .046 | .031 | .004 | .088 |
| VSE→PL→HW | .109 | .042 | .004 | .214 | .070 | .148 | -.025 | .166 |
| VSE→AC→HW | .056 | .047 | .001 | .112 | .062 | .025 | .008 | .117 |
| PL→AC→HW | .087 | .044 | .002 | .172 | .097 | .023 | .014 | .180 |
| PB→ALL→HWa | / | / | / | / | .019 | .051 | .000 | .039 |
| **Total Effectsb**  INT→HW | .022 | .764 | -.122 | .167 | -.024 | .735 | -.165 | .116 |
| VSE→HW | .192 | .011 | .043 | .340 | .134 | .066 | -.009 | .276 |
| PL→HW | .249 | <.001 | .123 | .375 | .207 | .001 | .084 | .331 |
| PB→HW | / | / | / | / | .269 | <.001 | .182 | .357 |

*Note*. a Sum of indirect effects of past behavior on behavior through all model constructs.

b Total effects comprising sums of all indirect effects through model constructs plus the direct effect. β = Standardized parameter estimate; 95% CI = 95% confidence interval of standardized parameter

estimate; LB = Lower bound of 95% CI; UB = Upper bound of 95% CI; MSE = motivational self-efficacy; INT = intention; RP = risk perception; ATT = attitude; SN = subjective norm; HK = health knowledge; HW = hand washing; VSE = volitional self-efficacy; PL = planning; AC = action control; PB = past behavior; →：indicating the former variable predicts the latter one; ∝: indicating the two variables correlating with each other.

Appendix C

*Standardized Parameter Estimates for the Direct, Indirect, and Total Effects in the Integrated Model of Mask Wearing Behavior Excluding and Including Past Behavior*

**Effects Model excluding past behavior Model including past behavior β *p* 95% CI β *p* 95% CI**

**LB UB LB UB**

**Direct Effects**

MSE→INT .563 <.001 .471 .655 .559 <.001 .465 .653

| RP→INT | .029 | .232 | -.019 | .077 | .028 | .250 | -.020 | .076 |
| --- | --- | --- | --- | --- | --- | --- | --- | --- |
| ATT→INT | .147 | <.001 | .083 | .212 | .147 | <.001 | .083 | .212 |
| SN→INT | .203 | <.001 | .122 | .284 | .205 | <.001 | .123 | .286 |
| HK→INT | -.001 | .972 | -.045 | .044 | -.003 | .891 | -.049 | .043 |
| HK→MW | .182 | <.001 | .095 | .269 | .121 | .007 | .033 | .209 |
| MSE→VSE | .881 | <.001 | .861 | .900 | .882 | <.001 | .857 | .907 |
| INT→PL | .393 | <.001 | .313 | .473 | .393 | <.001 | .313 | .473 |
| INT→AC | .262 | <.001 | .195 | .329 | .262 | <.001 | .196 | .328 |
| INT→MW | .099 | .287 | -.084 | .283 | .107 | .240 | -.071 | .285 |
| VSE→PL | .045 | .308 | -.042 | .132 | .044 | .317 | -.042 | .131 |
| VSE→AC | .307 | <.001 | .228 | .387 | .293 | <.001 | .213 | .373 |
| VSE→MW | -.126 | .109 | -.281 | .028 | -.137 | .074 | -.287 | .013 |
| PL→AC | .445 | <.001 | .381 | .509 | .439 | <.001 | .376 | .503 |
| PL→MW | .054 | .596 | -.145 | .253 | .076 | .442 | -.117 | .269 |
| AC→MW | .134 | .252 | -.095 | .363 | .043 | .710 | -.182 | .267 |
| PB ∝ MSE | / | / | / | / | .371 | <.001 | .297 | .446 |
| PB ∝ RP | / | / | / | / | .235 | <.001 | .154 | .317 |
| PB ∝ ATT | / | / | / | / | .285 | <.001 | .206 | .364 |
| PB ∝ SN | / | / | / | / | .258 | <.001 | .177 | .338 |
| PB ∝ HK | / | / | / | / | .338 | <.001 | .261 | .414 |
| PB→INT | / | / | / | / | .010 | .669 | -.037 | .057 |
| PB→PL | / | / | / | / | .016 | .471 | -.028 | .060 |
| PB→VSE | / | / | / | / | -.005 | .837 | -.049 | .039 |
| PB→AC | / | / | / | / | .051 | .003 | .017 | .084 |
| PB→MW | / | / | / | / | .250 | <.001 | .163 | .337 |
| **Indirect Effects**  MSE→INT→MW | .056 | .289 | -.048 | .160 | .060 | .243 | -.040 | .160 |
| RP→INT→MW | .003 | .427 | -.004 | .010 | .003 | .411 | -.004 | .010 |

| ATT→INT→MW | .015 | .301 | -.013 | .042 | .016 | .256 | -.011 | .043 |
| --- | --- | --- | --- | --- | --- | --- | --- | --- |
| SN→INT→MW | .020 | .299 | -.018 | .058 | .022 | .449 | -.016 | .059 |
| HK→INT→MW | .000 | .972 | -.005 | .004 | .000 | .892 | -.005 | .005 |
| MSE→VSE→MW | -.111 | .109 | -.248 | .025 | -.121 | .074 | -.253 | .012 |
| INT→PL→MW | .021 | .597 | -.057 | .099 | .030 | .443 | -.046 | .106 |
| INT→AC→MW | .035 | .257 | -.026 | .096 | .011 | .711 | -.048 | .070 |
| VSE→PL→MW | .002 | .638 | -.008 | .013 | .003 | .542 | -.007 | .014 |
| VSE→AC→MW | .041 | .257 | -.030 | .112 | .012 | .711 | -.053 | .078 |
| PL→AC→MW | .060 | .253 | -.043 | .162 | .019 | .711 | -.080 | .117 |
| PB→ALL→MWa | / | / | / | / | .006 | .445 | -.009 | .021 |
| **Total Effectsb**  INT→MW | .179 | .015 | .035 | .323 | .155 | .030 | .015 | .295 |
| VSE→MW | -.080 | .295 | -.230 | .070 | -.120 | .105 | -.265 | .025 |
| PL→MW | .113 | .178 | -.051 | .278 | .095 | .247 | -.065 | .254 |
| PB→MW | / | / | / | / | .256 | <.001 | .170 | .343 |

*Note*. a Sum of indirect effects of past behavior on behavior through all model constructs.

b Total effects comprising sums of all indirect effects through model constructs plus the direct effect. β = Standardized parameter estimate; 95% CI = 95% confidence interval of standardized parameter

estimate; LB = Lower bound of 95% CI; UB = Upper bound of 95% CI; MSE = motivational self-efficacy; INT = intention; RP = risk perception; ATT = attitude; SN = subjective norm; HK = health knowledge; MW = mask wearing; VSE = volitional self-efficacy; PL = planning; AC = action control; PB = past behavior; →：indicating the former variable predicts the latter one; ∝: indicating the two variables correlating with each other.

Appendix D

*Standardized Parameter Estimates for the Direct, Indirect, and Total Effects in the Integrated Model of Social Distancing Behavior Excluding and Including Past Behavior*

**Effects Model excluding past behavior Model including past behavior β *p* 95% CI β *p* 95% CI**

**LB UB LB UB**

**Direct Effects**

| MSE→INT | .639 | <.001 | .563 | .716 | .640 | <.001 | .562 | .718 |
| --- | --- | --- | --- | --- | --- | --- | --- | --- |
| RP→INT | .042 | .091 | -.007 | .091 | .043 | .089 | -.007 | .092 |
| ATT→INT | .158 | <.001 | .093 | .233 | .158 | <.001 | .093 | .222 |
| SN→INT | .093 | .009 | .023 | .164 | .092 | .011 | .021 | .162 |
| HK→INT | .022 | .304 | -.020 | .065 | .026 | .247 | -.018 | .070 |
| HK→SD | .236 | <.001 | .153 | .318 | .192 | <.001 | .106 | .277 |
| MSE→VSE | .894 | <.001 | .877 | .911 | .896 | <.001 | .875 | .917 |
| INT→PL | .099 | .214 | -.057 | .256 | .106 | .177 | -.048 | .260 |
| INT→AC | -.084 | .136 | -.195 | .026 | -.082 | .147 | -.192 | .029 |
| INT→SD | .015 | .861 | -.153 | .183 | .025 | .772 | -.141 | .190 |
| VSE→PL | .801 | <.001 | .637 | .965 | .771 | <.001 | .607 | .934 |
| VSE→AC | .720 | <.001 | .623 | .817 | .721 | <.001 | .623 | .819 |
| VSE→SD | .009 | .915 | -.162 | .181 | .004 | .960 | -.165 | .174 |
| PL→AC | .401 | <.001 | .325 | .477 | .392 | <.001 | .315 | .469 |
| PL→SD | .076 | .278 | -.061 | .212 | .055 | .424 | -.080 | .190 |
| AC→SD | .179 | .049 | .001 | .358 | .158 | .079 | -.018 | .335 |
| PB ∝ MSE | / | / | / | / | .303 | <.001 | .225 | .382 |
| PB ∝ RP | / | / | / | / | .213 | <.001 | .131 | .295 |
| PB ∝ ATT | / | / | / | / | .216 | <.001 | .134 | .298 |
| PB ∝ SN | / | / | / | / | .211 | <.001 | .128 | .293 |
| PB ∝ HK | / | / | / | / | .355 | <.001 | .280 | .431 |
| PB→INT | / | / | / | / | -.001 | .964 | -.048 | .045 |
| PB→PL | / | / | / | / | .069 | .030 | .007 | .131 |
| PB→VSE | / | / | / | / | -.008 | .710 | -.048 | .033 |
| PB→AC | / | / | / | / | .016 | .481 | -.028 | .059 |
| PB→SD | / | / | / | / | .155 | <.001 | .070 | .240 |
| **Indirect Effects**  MSE→INT→SD | .010 | .861 | -.098 | .117 | .016 | .772 | -.090 | .122 |
| RP→INT→SD | .001 | .862 | -.007 | .008 | .001 | .775 | -.006 | .008 |

| ATT→INT→SD | .002 | .861 | -.024 | .029 | .004 | .772 | -.022 | .030 |
| --- | --- | --- | --- | --- | --- | --- | --- | --- |
| SN→INT→SD | .001 | .862 | -.014 | .017 | .002 | .773 | -.013 | .018 |
| HK→INT→SD | .000 | .863 | -.003 | .004 | .001 | .778 | -.004 | .005 |
| MSE→VSE→SD | .008 | .915 | -.145 | .162 | .004 | .491 | -.148 | .156 |
| INT→PL→SD | .007 | .414 | -.010 | .025 | .006 | .491 | -.011 | .023 |
| INT→AC→SD | -.015 | .235 | -.040 | .010 | -.013 | .263 | -.036 | .010 |
| VSE→PL→SD | .061 | .281 | -.050 | .171 | .043 | .426 | -.062 | .147 |
| VSE→AC→SD | .129 | .051 | -.001 | .259 | .114 | .081 | -.014 | .243 |
| PL→AC→SD | .072 | .053 | -.001 | .145 | .062 | .083 | -.008 | .132 |
| PB→ALL→SDa | / | / | / | / | .009 | .154 | -.003 | .021 |
| **Total Effectsb**  INT→SD | .015 | .867 | -.155 | .184 | .024 | .776 | -.142 | .190 |
| VSE→SD | .257 | .004 | .080 | .433 | .209 | .018 | .036 | .382 |
| PL→SD | .148 | .026 | .018 | .277 | .117 | .074 | -.011 | .246 |
| PB→SD | / | / | / | / | .164 | <.001 | .079 | .248 |

*Note*. a Sum of indirect effects of past behavior on behavior through all model constructs.

b Total effects comprising sums of all indirect effects through model constructs plus the direct effect. β = Standardized parameter estimate; 95% CI = 95% confidence interval of standardized parameter

estimate; LB = Lower bound of 95% CI; UB = Upper bound of 95% CI; MSE = motivational self-efficacy; INT = intention; RP = risk perception; ATT = attitude; SN = subjective norm; HK = health knowledge; SD

= social distancing; VSE = volitional self-efficacy; PL = planning; AC = action control; PB = past behavior; →：indicating the former variable predicts the latter one; ∝: indicating the two variables correlating with each other.
